# Ten-year results of quality assurance in radiotherapy chart round

**DOI:** 10.1186/1472-6963-13-148

**Published:** 2013-04-23

**Authors:** Bardia Taghavi Bayat, Suki Gill, Shankar Siva, Keen Hun Tai, Michael Lim Joon, Farshad Foroudi

**Affiliations:** 1 Bendigo Base Hospital, Bendigo, Australia; 2 Department of Radiation Oncology, Peter MacCallum Cancer Centre, Locked Bag 1, A’Beckett Street, East Melbourne, VIC 8006, Australia; 3 The University of Melbourne, Melbourne, Australia

**Keywords:** Audit, Radiotherapy, Urology, RANZCR, Quality

## Abstract

**Background:**

The Royal Australian and New Zealand College of Radiologists (RANZCR) initiated a unique instrument to audit the quality of patient notes and radiotherapy prescriptions. We present our experience collected over ten years from the use of the RANZCR audit instrument.

**Methods:**

In this study, the results of data collected prospectively from January 1999 to June 2009 through the audit instrument were assessed. Radiotherapy chart rounds were held weekly in the uro-oncology tumour stream and real time feedback was provided. Electronic medical records were retrospectively assessed in September 2009 to see if any omissions were subsequently corrected.

**Results:**

In total 2597 patients were audited. One hundred and thirty seven (5%) patients had one hundred and ninety nine omissions in documentation or radiotherapy prescription. In 79% of chart rounds no omissions were found at all, in 12% of chart rounds one omission was found and in 9% of chart rounds two or more omissions were found. Out of 199 omissions, 95% were of record keeping and 2% were omissions in the treatment prescription. Of omissions, 152 (76%) were unfiled investigation results of which 77 (51%) were subsequently corrected.

**Conclusions:**

Real-time audit with feedback is an effective tool in assessing the standards of radiotherapy documentation in our department, and also probably contributed to the high level of attentiveness. A large proportion of omissions were investigation results, which highlights the need for an improved system of retrieval of investigation results in the radiation oncology department.

## Background

The aim of clinical audit is to proactively seek out shortcomings in the medical system, recommend improvements to clinical service and ultimately enhance the quality of patient care [1]. In 1999, the Royal Australian and New Zealand College of Radiologists (RANZCR) designed an instrument to check and record the quality of radiotherapy notes and prescriptions [2]. This audit tool has been shown to be cost effective, to improve targeted performance and positively received by participants [3]. The RANZCR audit tool mandates feedback to treating clinicians as an educational function as well as to provide an additional tier of safety assessment. Studies assessing the impact of implementation of audit instruments document improved consultant practice based on these predetermined targets [4]. It is also an opportunity for clinicians to have ongoing support and discussion with their radiation oncology peers with regards to patient management.

A Cochrane review of audit with feedback looking at 118 studies found that the absolute compliance rate improvement could be as high as +68% or as low as −10% compared to control [5]. Although the RANZCR audit instrument has been in use for over 10 years, the standards at which radiation oncology centers are practicing over a long period of time have not been benchmarked in Australasia. The primary objective of this study is to assess the frequency of errors and omissions picked up by the audit instrument in 10 years of clinical practice in a large academic cancer centre. The secondary objective of this study is to assess the rate of corrections of any omissions detected during audit following a real- time feedback system.

## Methods

This was a retrospective study of all patients with urological cancers who underwent radical radiotherapy treatment between 28.1.1999 to 25.6.2009 and audited at chart round. Analysis was performed using data collection sheets prospectively completed during this time period. Live feedback was provided during the audit if the consultant being audited was present, or at a later date if not. At the end of each chart round during this period, the omissions detected at audit were collected onto a separate data collection sheet. An omission is defined as a criterion that is not met in the audit tool checklist.

The uro-oncology radiotherapy chart round at our centre is held weekly. It is attended by radiation therapists involved in planning, the radiation oncologists, radiation oncology fellows and radiation oncology registrar; and from time to time depending on the clinical need, a medical physicist would be present. At chart round, the management and contouring of all patients about to undergo radiotherapy is thoroughly discussed. However, only new cases of radical dose radiotherapy are audited using the RANZCR tool. For each patient the audit was conducted after the radiotherapy planning process but before or during the first week of radiotherapy treatment. Patients to be audited were randomly allocated by a senior radiation therapist to one of the auditors present. The auditor was part of the uro-oncology unit, but not directly involved in the care of the patient. He or she could be a registrar who had completed one year of training and the part 1 FRANZCR exam and supervised by a clinical fellow or a consultant radiation oncologist. The auditor would review the patient’s notes; identify relevant imaging, pathology and planning charts whilst completing the appropriate checklist on the RANZCR audit score sheet.

The RANZCR audit instrument is available on the RANZCR website [6]. The instrument was refined following recommendations by Toohey et al. published in 2008 [7]. In summary, the audit questions were broadly similar; but changes were made to demonstrate that both documentation in notation of the case notes and appropriate filing of required histology and imaging occurred, and two separate criteria for target coverage and critical structure doses were added.

Omissions were defined as a criteria or question that was marked as not meeting the stipulated gold standard set by the RANZCR audit tool during each chart round audit session.

In patients with omissions, electronic medical records (Verdi™;IP Health, Australia) were reviewed in September 2009 to check that the omissions detected by the audit tool had been updated subsequent to the audit. We have assumed for this study that omissions detected in the prescription sheet were corrected at chart round. The frequency of each piece of missing information, type of information, and whether it was subsequently corrected was imputed and analyzed in Excel™ (Microsoft Corporation, Redmond, WA).

## Results

From January 1999 to June 2009, 2597 patients were audited. A median of five people were present during chart round each week (interquartile range 2 to 8). There was a median of 4 patients discussed at each chart round (range 1 to 15).

Table 1 illustrates the omissions and rate of subsequent corrections. In 79% of chart rounds no omissions were found at all. However in 12% of chart rounds one omission was found and in 9% of chart rounds two or more omissions were found.

**Table 1:** Number of omissions for each criterion; and number corrected subsequently out of 2597 patients

		**Number of omissions**	**Number corrected subsequently**
**Departmental or hospital clinical record**			
1	History recorded	23	9
2	Examination recorded	3	1
3	Primary tumour site correctly documented	0	0
4	Histology correctly documented and report filed	45	29
5	Relevant imaging of treated site correctly documented and reports filed	105	48
6	Tumour stage correctly documented	5	2
7	Rationale for radiotherapy documented	0	0
8	Treatment intent documented	0	0
9	Discussion of treatment risks and consent recorded	0	0
10	Letter or notes copied to referring doctor	8	8
Treatment prescription			
11	Legible patient name is present on all prescriptions	0	0
12	Treatment site specified and correct	0	0
13	Laterality (i.e. ‘left’ or ‘right’) for treated site correctly documented	1	1
14	Radiation modality and energy for all phases	0	0
15	Total dose specified for all phases	0	0
16	Dose per fraction specified for all phases	0	0
17	RT dose point specified for all phases	3	3
18	Number of fractions/day specified for all phases	0	0
19	Number of fractions per week for all phases	0	0
20	Treatment prescriptions are signed/approved and dated	0	0
**Simulation and planning**			
21	Isodoses/treatment plan signed/approved and dated by doctor	0	0
22	Legible and correct patient name on all simulation/VP film/electronic images	0	0
23	Simulation image is signed or approved and dated by doctor	0	0
**Performance criteria**			
24	Indication for treatment	0	0
25	Treatment intent (radical vs. palliative)	0	0
26	Target volume coverage	0	0
27	Critical structure doses	0	0
28	Prescribed total dose for each volume	0	0
29	Fractionation schedule	0	0
30	Clinical protocol or practice guideline followed	0	0
31	Research or Study followed	0	0
	(*for 6 patients the actual omission was unclearly documented).	6	0
Total		199	101

One hundred and ninety nine omissions occurred in 137 patients (i.e. 5% of all patients audited) over 10.5 years. Of these omissions, 105 were inadequate documentation or filing of investigation results (53%). A further 45 (23%) were inadequate documentation or filing of the histology report.

The third most frequent omission was in the adequate recording of the medical history, of which there were 23 omissions. Only 9 of these were subsequently corrected. There were 8 patients missing a letter to the referring doctor at chart round, but all 8 letters to the referring doctor were subsequently sent. Other omissions were infrequent, including five missing tumour stage, three were missing a patient clinical examination record, one prescription did not specify the laterality of the tumour, and three prescriptions did not specify the prescription dose point. Two of five missing tumour stage were corrected, one of three missing the patient clinical examination record was corrected, and all missing documentation in the radiotherapy prescription were subsequently corrected.

Each omission was assessed individually and none of these omissions would have been considered clinically serious, or dangerous to the patient. Overall, 75% of omissions were lack of documentation of investigations, either radiology or pathology. However, overall only 101 of 199 omissions detected at chart round were subsequently corrected (51% of all omissions), when checked in September 2009.

## Discussion

In our study, of the 2597 radically treated patients, the rate of omissions in radiotherapy prescription and clinical documentation, including filing of radiology and pathology source documentation was low. We found a rate of 20 omissions per year on average affecting 5% of all patients audited. Previous studies have shown that the audit and feedback tool improves clinician compliance with the criteria being audited [8]. The low rate of omissions is possibly because consultants are mindful that documentation is being monitored constantly, and therefore strive for a high standard in note keeping. It is likely therefore that the low rate of omissions was a result of the audit and feedback tool itself.

The highest rate of omission was in the filing of investigation results, which comprised 53% of all omissions. The likely cause for this shortcoming is that our centre is a tertiary centre and the majority of investigations would have been performed at another hospital and be stored elsewhere. It is unclear to what degree that retrieval of these investigation results was necessary in the management of these patients. Knowing the quality of life outcomes of the omissions had they been not detected would have been useful. The effect of an omission is very complex and difficult to measure. However none of the omissions that were analyzed resulted in a significant negative clinical outcome for the patient. It is likely that clinicians were aware of relevant investigations required to make clinical decisions and that these investigations were sighted even though they were not filed in the notes; and therefore investigation results not filed in the notes did not impact on patient care. Reviewing source documentation in oncology is very important, as it prevents the propagation of transcription errors. Given that the filing of source investigation reports comprised the highest rate of non-compliance with the audit tool, solutions to improve this aspect of clinical workflow are required. The retrieval of results was not much under the control of the radiation oncology department but largely the domain of the Health Information Services (HIS). There is a team effort required in that the clerks, receptionists and nurses track down all pathology and imaging referral details when the patient is seen in clinic, but once found, it is then someone else’s responsibility to ensure that the data gets into the patients’ notes, or is scanned onto electronic records. Often the people involved are based at different hospitals or at different sites. Our study highlights that there are potential weak links in this system, and perhaps also in other radiation oncology centres. For example in another large Australian oncology centre, Boxer et al. noted that scanning of histology and medical imaging reports did not occur in up to a third of cases [8]. An improved system of retrieval and filing of investigation results throughout the health information service is required. Possible suggestions include the more efficient use of departmental secretarial time, or in the future, a centralized inter-hospital electronic patient database.

The lack of a medical history in the electronic medical records at the time of chart round was not unexpected. Due to secretarial constraints, there may have been a delay in typing up the medical history at the time of chart round. However, it is interesting that only nine of 23 of these missing notes were subsequently typed up. Based on anecdotal experience, we believe that this phenomenon may have occurred due to the assumption that missing clinical histories were attributable to delays in typing, and hence not followed up by clinicians. It may have been that only some of the missing histories were due to delays in typing but in the absence of subsequent verification by the designated clinician(s), a small number of dictated narrative histories were not typed. This is not to say that documentation of the consultation was not made in these cases, as notes may have been made in the hardcopy paper notes. This is a limitation of our study, for example, if the patient were an inpatient seen on the ward, the medical history would be handwritten in the notes, whereas in this study we only assessed the electronic records retrospectively. In addition, all patients in this audit had letters sent to the referring doctor, which usually does outline the medical history and management plan.

Only four omissions in the radiotherapy prescription were found during chart round. The reason for the low level of omissions in the prescription is because the prescription is created from a standard electronic template, initially checked by two separate radiation therapists, followed by the radiation oncologist at the time of providing final authorization. By the time it is assessed at chart round the prescription is being checked for its fourth time. No changes in the management of the patient, i.e. performance criteria, were found, because at each chart round the patients are discussed as a group by the radiation oncologists after target delineation but prior to being planned. Therefore by the time the performance criteria are audited at chart round, the management and target delineation of the patient had already been discussed and agreed upon by the group. None of the omissions in prescription documentation were considered harmful to the patient because the actual three-dimensional radiotherapy plan was assessed and approved separately on a radiotherapy planning terminal and there were no errors in this section noted.

The aim of auditing is to provide reassurance that the best quality of service is being achieved using the available resources, while emphasizing opportunities for improvement and a mechanism for change [7]. In the single machine unit trial, Shakespeare et al. looked at 130 patients selected randomly across four sites and found that at hub sites 79.6% of criteria were adequate and at the rural sites 84.4% of criteria were adequate [9]. In another radiation oncology department with ten radiation oncologists, only 170 out of 208 patients (81.7%) had complete audit assessments over the period of one year [8]. The findings of our study, whereby only 5% of patients have non-adherence to the audited criteria, are better than the findings from some other studies utilizing the RANZCR audit instrument. This is possibly because the instrument has been in use continuously in our department for over ten years for over 2597 patients, and radiation oncologists and fellows at our centre are expecting to be audited, and therefore attempt to keep a high standard of documentation. This is in keeping with results by Leong et al. who compared results of chart audit with feedback in 36 patients in the first 6 months of a quality improvement program, and in 39 patients in the second six months [4]. Protocol adherence improved in this period from 90.3% to 96.6% in that study. One method that could improve on compliance would be to use the tool at the time the patient is first seen in clinic as a prompt for the necessary documentation.

## Conclusion

Our report has found that the chart round meetings are an efficient way to discuss and improve on the documentation and management of patients undergoing radiotherapy for urological malignancies. The audit tool contributed to an efficient process of high quality note keeping. The findings of this study suggest that an effective policy for the retrieval and incorporation of investigation results needs to be implemented, preferably nationally.

## Competing interests

The authors declare that they have no competing interests.

## Authors’ contributions

FF designed the study. BT collected and analyzed the data and prepared the first manuscript draft. SG, SS, MLJ and KHT assisted with preparation of the manuscript. All authors read and approved the final manuscript.

## Pre-publication history

The pre-publication history for this paper can be accessed here:

http://www.biomedcentral.com/1472-6963/13/148/prepub
